# Exposure-in-vivo containing interventions to improve work functioning of workers with anxiety disorder: a systematic review

**DOI:** 10.1186/1471-2458-10-598

**Published:** 2010-10-11

**Authors:** Erik Noordik, Jac JL van der Klink, Elmer F Klingen, Karen Nieuwenhuijsen, Frank JH van Dijk

**Affiliations:** 1 Coronel Institute of Occupational Health, Academic Medical Center (AMC), University of Amsterdam, P.O. Box 22700, 1100 DE Amsterdam, The Netherlands; 2 University Medical Center Groningen, Department of Health Sciences, Groningen, The Netherlands; 3 Coronel Institute of Occupational Health, Academic Medical Center (AMC), University of Amsterdam, Amsterdam, The Netherlands

## Abstract

**Background:**

Anxiety disorders are associated with functional disability, sickness absence, and decreased productivity. Effective treatments of anxiety disorders can result in remission of symptoms. However the effects on work related outcomes are largely unknown. Exposure in vivo is potentially well fit to improve work-related outcomes. This study systematically reviews the effectiveness of exposure-in-vivo containing interventions in reducing work-related adverse outcomes in workers with anxiety disorders.

**Methods:**

A systematic study search was conducted in Medline, Cinahl, Embase and Psycinfo. Two reviewers independently extracted data and from each study assessed the quality of evidence by using the GRADE approach. We performed a meta-analysis if data showed sufficient clinical homogeneity.

**Results:**

Seven studies containing 11 exposure-in-vivo interventions were included. Four studies were focused on Obsessive Compulsive Disorder (OCD), two on Post Traumatic Stress Disorder (PTSD), and one on a mixed group of OCD and severe phobias. The studies were grouped according to type of anxiety disorder and subsequently according to type of comparisons. For OCD, exposure-in-vivo containing interventions can yield better work-related outcomes compared to medication (SSRIs) and relaxation but not better compared to response prevention. The results on anxiety outcomes were similar. The net contribution of exposure in vivo in two OCD intervention programs is also presented as a meta-analysis and shows significant positive results on work role limitations. The calculated pooled effect size with 95% confidence interval was 0.72 (0.28, 1.15). For PTSD, exposure-in-vivo containing interventions can yield better work-related and anxiety-related outcomes compared to a waiting-list but not better compared to imaginal exposure.

**Conclusions:**

Exposure in vivo as part of an anxiety treatment can reduce work-related adverse outcomes in workers with OCD and PTSD better than various other anxiety treatments or a waiting-list. We recommend that it should be studied how the results of these studies can be transferred to the practice of occupational health professionals and how clinicians can make better use of them to improve work-related outcomes. In future research, priority should be given to high-quality randomised controlled trials (RCTs) in which exposure-in-vivo containing interventions are applied to a variety of anxiety disorders and compared with other clinical anxiety treatments such as SSRIs. Work-related outcomes, in particular work functioning and sickness absence, need to be assessed with reliable and valid measures.

## Background

Anxiety disorders have a substantial impact on personal lives, companies, and society and are highly prevalent in the general population. The twelve-month prevalence rates among the general adult population in Canada, the US, and Europe are 12%, 18%, and 12%, respectively [1-3]. These disorders are associated with functional disabilities in the social, emotional, and physical domains of life which also affect work [4-6]. Effects on work are increased unemployment rates, increased sickness absence, and decreased productivity [6-11]. The annual costs of anxiety disorders impose a considerable financial burden on American and European societies, the bulk of which are due to reduced working capacity or early retirement [3,12,13]. It is postulated that a substantial part of these costs can be avoided with greater recognition and appropriate interventions [6].

Cognitive behavioural therapy (CBT), pharmacotherapy, or a combined treatment of CBT and medication are effective in symptom reduction for a variety of anxiety disorders [14-23]. Recovery in social functioning, including work functioning, is an aspect of recovery that often does not occur completely or takes more time to manifest than other symptoms, as has been described for psychiatric symptomatology in general, depressive symptoms and OCD symptoms [4,14,24-26]. This suggests that more attention paid to return-to-work during regular treatment or the introduction of specific work-directed interventions in addition to clinical treatment might be advantageous.

Exposure in vivo, which is a common behavioural component of CBT for different anxiety disorders, might be promising for reducing work-related adverse outcomes. Exposure in vivo can be aimed directly at behavioural change in dealing with anxiety-provoking work situations during return-to-work [27]. Exposure in vivo provides the opportunity for workers to learn to deal gradually with anxiety-provoking work situations during return-to-work or actual work functioning. Other types of exposure, such as imaginal or interoceptive exposure, are more indirectly aimed at a real life confrontation with anxiety-provoking work situations. Imaginal exposure, for example, is aimed at cognitive restructuring and can be used in preparation for a real life confrontation with anxiety-provoking situations. Furthermore, exposure in vivo is not complicated by the adverse side effects common in pharmacotherapy, which can even result in decreased work functioning [28,29].

We considered four dimensions of work functioning for workers with CMDs relevant as potential effects of exposure in vivo [30]. One dimension describes the economic aspects related to the output of workers such as in terms of loss of productivity due to sick leave and associated costs. A second dimension represents the process of work functioning by assessing the work role limitations of workers. A third dimension explicates the quality of work e.g. in terms of errors or a risk of accidents. A fourth dimension considers the personal effort necessary to remain productive such as in terms of extra effort days.

As a recent overview of reducing work-related adverse effects of exposure in vivo in treatment programmes for different anxiety disorders is lacking, we aimed to review these effects systematically. We reviewed studies that included exposure in vivo as part of a treatment programme and compared this approach with either a programme without exposure in vivo, a waiting list condition, placebo treatment, or care as usual. Our main interest was to determine whether a treatment programme that included exposure in vivo resulted in better work-related and anxiety-related outcomes than a treatment programme without exposure in vivo. More knowledge of the work-related adverse effects of exposure in vivo in anxiety treatment could support decision making regarding optimal treatment for workers.

## Methods

### Search method for identification of studies

A systematic search was conducted in four electronic databases: MEDLINE (Ovid 1966-2007), CINAHL (1982-2007), EMBASE (1980-2007), and PsycINFO (Silverplatter 1972-2007). The search was subsequently extended by checking the references of all retrieved reviews and the references, citations, and authors of all included articles.

Four groups of free text words in the title, abstract, or full text of the article were combined with AND: i.e., we combined words related to anxiety, exposure, and work with words referring to controlled or randomised controlled studies.

1. Type of participants (anxiety-related words): fear* OR fright* OR afraid* OR angst* OR agoraphobia OR GAD OR OCD OR Phobia OR PTSD OR worr* OR panic* OR obses* OR intrusive OR irratio* OR preoccupation OR ruminat* OR compuls* OR catas* OR escap* OR avoid* OR anx*.

2. Type of intervention (exposure-related words): vivo* OR reinf* OR habitu* OR exti* OR conditioni* OR Skinner OR behav* OR stimulus* OR expos*.

3. Type of research design (words related to controlled and randomised controlled studies): clinical trial OR Clinical trials OR (clin* adj25 trial*) OR placebos OR placebo* OR random* OR evaluation studies OR prospective studies OR (control* or prospective* or volunteer*) OR ((singl* OR doubl* OR tripl* OR trebl*) adj25 (blind* OR mask* OR dummy)).

4. Type of outcome (work-related words): job performance OR job re-entry OR employment OR (rehabilitation, vocational) OR sick leave OR work OR disability evaluation OR Occupational Therapy OR return-to-work OR occupational therap* OR occupational intervention* OR supported employment OR vocational rehabilitation OR work capacity evaluation OR vocational guidance OR absenteeism OR occupational health services OR occupational health OR unemployed OR employed OR unemployment OR sick* absence OR retirement OR disability pension OR occupation* OR job OR vocational.

The selected words are partly database-specific (see Additional file 1: Searchstrings). The work-related words in section four are mostly selected from a study by Haafkens et al. on work-related terms in searching for literature on chronic diseases (rheumatoid arthritis, diabetes mellitus, hearing problems, and depression) [31]. The work-related words: work* and occupation* are sensitive single terms used to locate occupational health studies as advocated by Verbeek et al. [32]. Furthermore, to identify studies with the appropriate design, we selected words related to controlled and randomised controlled trials from the search strategy of the Cochrane Collaboration Depression, Anxiety, and Neurosis Group (CCDAN).

### Criteria for inclusion/exclusion and procedure

We included studies that met the following five criteria:

1. Participants were selected for the original study because of their anxiety disorder or anxiety complaints, and were aged between 18 and 65 years.

2. Exposure in vivo was used explicitly as a central component of therapy, and was performed gradually. We excluded studies in which the central part of therapy consisted of only exposure in vitro, imaginal exposure, or interoceptive exposure.

3. The 'exposure in vivo' intervention was compared with another intervention aimed at diminishing anxiety, such as anxiolytic or antidepressant medication, cognitive behavioural psychotherapy without exposure, a waiting-list treatment, imaginal or interoceptive exposure, a placebo, or care as usual.

4. One or more outcome parameters were related to work functioning, where work functioning includes employment status, absenteeism, and presenteeism. Other than unemployment, relevant work-related outcomes can be absenteeism measures such as duration or frequency of sick leave, or measures of functioning while at the workplace such as the Work scale of the Social Adjustment Scale (SAS) and the Sheenan Disability Scale (SDS).

5. The study design was a randomised controlled trial (RCT) or a clinical controlled trial (CCT). We also included cluster randomised controlled trials.

Three reviewers (EN, KN, and EK) conducted the initial selection from the studies identified based on title and/or abstract. The second selection was performed by two independent reviewers (EN and EK/KN) who systematically applied the inclusion and exclusion criteria to the full-text articles. Disagreements between the two reviewers were discussed until consensus was reached. If no consensus could be reached, a third reviewer (KN/EK) was consulted, and acted as a referee.

### Methodological quality

After inclusion, two independent reviewers (EN and KN) independently estimated the quality of evidence of each study that was included. To judge the quality of evidence we used the GRADE criteria [33]. In this approach, the quality of evidence is based on a judgement of the limitations in the design (i.e., risk of bias), the indirectness of evidence, unexplained heterogeneity or inconsistency of results, imprecision of results, and probability of publication bias, respectively. The quality of evidence can be categorised as high, moderate, low, and very low. A high quality of evidence is associated with randomised trials with a low risk of bias. In cases of unclear or high risk of bias the quality of evidence was downgraded one level, from high to moderate, if the study limitations were judged to be serious. In case of a high risk of bias the quality of evidence was downgraded two levels, from high to low, if study limitations were judged very serious. The assessment of the risk of bias is based on a judgement of the sequence generation, allocation concealment, blinding of outcome assessors, incomplete outcome data, selective outcome reporting, and other sources of bias, respectively [33]. Blinding of participants and health care providers were not used as a criterion, as these aspects are not adequately applicable in RCTs in the field of occupational health research [34,35]. The risk of bias was finally judged as high, unclear, or low. Study limitations (i.e., risk of bias) was the most relevant criterion to judge the quality of evidence of the studies in this review. The other four criteria were hardly applicable to the included studies.

### Data analysis

From the studies with multiple intervention groups, we selected groups for which exposure in vivo was an important part of the intervention (intervention groups) and compared the outcome scores of these groups with the outcome scores of groups without exposure (control groups). We considered two types of outcome scores: work-related and anxiety-related outcomes. A study was considered to have found positive results when the treatment programme of the intervention group improved the outcome score significantly more than the treatment programme of the control group did (significant positive = sp). Studies that found no significant differences between the intervention and control groups reported neutral results (not significant = ns). Studies which found a significantly worse score for the intervention group compared with the control group were considered to have produced negative results (significant negative = sn).

If studies had more than one experimental exposure group, we considered each experimental group with a different treatment programme as a separate group. If studies reported multiple anxiety outcomes for a single experimental group, we summarised the effects on different outcomes by calculating the sum of all positive, neutral, and negative outcomes.

Whenever significant or non-significant effects were reported, we calculated the effect size using standardised mean differences (SMDs), as supported by the available data. The SMDs were based on the final post-treatment scores and were calculated with a fixed or random-effect model [36]. These SMDs are also known as 'Hedges' (adjusted) g'. Whenever the statistical significance calculated differed from that reported by the authors, the calculated SMD was used in the evidence synthesis. For uniformity of reporting, any improved work-related or anxiety-related effect that was represented by a negative value was transformed by multiplying the mean negative SMD by -1. SMDs of 0.2 or less are considered a small effect, SMDs of 0.2 or more are considered a medium-sized effect, and SMDs of more than 0.8 are considered a large effect [36]. Whenever SMDs and the 95% confidence interval were calculated for multiple comparisons in one study, we used half of the number of workers that were reported in the study for the control group (N/2), in order to prevent a unit-of-analysis error due to double counting participants [36].

Whenever selected workers, intervention groups, control groups, and outcomes were considered clinically heterogeneous, we presented the results separately. Whenever selected workers, intervention groups, control groups, and outcomes were considered sufficiently clinically homogenous, we planned to perform a meta-analysis. In addition, in order to study the net contribution of exposure in vivo, we planned to perform a meta-analysis of studies in which the only difference between groups was the application of exposure in vivo, regardless of the content of other components of the intervention (e.g., exposure in vivo included in group CBT plus SSRIs compared to only SSRIs, as opposed to exposure in vivo at home compared to response prevention). We calculated a pooled effect size by pooling SMDs. between groups using a fixed-effect model. Statistical heterogeneity between experimental groups was tested with the I^2 ^test. If the calculated I^2 ^score was lower than 50%, we considered the pooled effect sizes to be sufficiently statistically homogenous. If I^2 ^was greater than 50%, we considered the effect sizes to be statistically heterogeneous and we recalculated the pooled effect size with a random-effects model [36].

## Results

**Figure 1:**
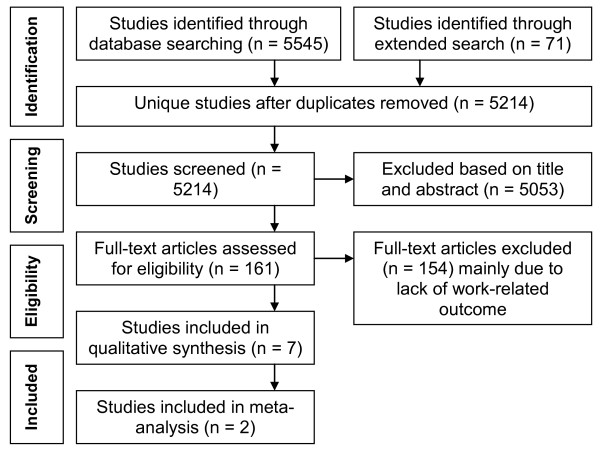
**Flowchart of the inclusion and exclusion during the searching process**.

A total of 5,545 publications were retrieved from our systematic search of four databases. We identified 402 duplications, leaving 5,143 unique studies. Applying the eligibility inclusion criteria to the title and abstract eliminated 5,053 studies. Applying the eligibility inclusion criteria to the 90 remaining full-text articles left only three relevant articles. The extended search resulted in the selection of another 71 relevant studies, which were read in full. Another four studies from this group could be included, resulting in a total of seven included studies (See Figure 1 for a flowchart of the inclusion process). The main reason for exclusion of full text articles was the absence of a specific work-related outcome.

### Characteristics of included studies

Additional file 2 shows the characteristics of the seven included studies. Four studies had two experimental groups with different interventions and a common control group. The other three studies had single experimental and control groups. We present the characteristics of all experimental groups separately. Three studies were published before 2000 and four were published since 2000. Participants in four of the included studies had an obsessive-compulsive disorder (OCD) for many years (mean range 9.8-22 years). In one study, participants had OCD or a severe phobia (mean duration 8 years). In two studies, participants had post-traumatic stress disorder (PTSD). The age of the participants in the studies ranged from 15 to 80 years. Three studies reported the percentage of participants working at baseline. Four studies were randomised controlled trials and three were controlled trials. Follow-up periods were between 8 and 17 weeks for three studies, 26 weeks for one study, and 12 months for three studies. The total number of participants in the experimental and control groups ranged from 6 to 74 and 5 to 75, respectively. Four studies had been conducted in the USA, two in the UK, and one in Austria. In two studies, workers with OCD were treated at a university clinic. In the other studies, workers with OCD were treated in an outpatient setting, an out- or inpatient setting, or the setting was not reported. In one study, workers with PTSD were treated in an academic or community clinic, and in the other study workers with PTSD were treated in an academic clinic.

The treatment programme of the OCD and PTSD intervention groups consisted mainly of exposure in vivo. In three intervention groups with OCD, exposure in vivo was combined with medication therapy (SSRIs or clomipramine) or response prevention. In one intervention group with PTSD, exposure in vivo was combined with cognitive restructuring. The treatment programmes for the OCD and PTSD control groups all consisted of a treatment without exposure in vivo. However, the content of this treatment varied. Workers with OCD in control groups received SSRI medication, systematic self-relaxation, clomipramine medication with anti-exposure homework, marital therapy, or response prevention. Workers with PTSD in the control group received wait-list or imaginal exposure. Additional file 3 shows a more detailed description of the components, duration, and provider of the treatment programmes in the experimental and control condition of each study.

Two studies of OCD and one study of PTSD used the Work and Social Adjustment Scale as a work-related outcome measure; the other four studies each used other work-related outcome measures. Two studies of OCD used the same anxiety-related outcome measure (Y-BOC); the other three studies of OCD used multiple measures for anxiety, obsessions, and compulsive behaviour. The two studies of PTSD used various anxiety-related outcome measures.

### Methodological quality

Additional file 4 shows the judgement of the quality of evidence for the seven included studies and one meta-analysis. In the risk-of-bias assessment, we found that one OCD study and one PTSD study had a high risk of bias and subsequently a low quality of evidence [37,38]. Three OCD studies were found to have an unclear risk of bias and subsequently a moderate quality of evidence [39-41]. One OCD and one PTSD study had a low risk of bias and subsequently a high quality of evidence [42,43].

### Results of data and quality of evidence

**Figure 2:**
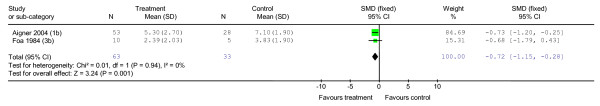
**Meta-analysis of work-related effect of net contribution of exposure in vivo based on two OCD studies**.

**Figure 3:**
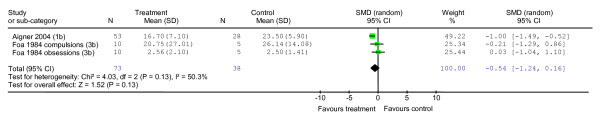
**Meta-analysis of anxiety-related effect of net contribution of exposure in vivo based on two OCD studies**.

Additional file 5 shows the calculated effect sizes on the work-related outcomes for OCD and PTSD, respectively, from the studies that were included in the different comparisons. In Figure 2, we present the calculated pooled effect size of a meta-analysis of two studies concerning work-related outcomes in workers with OCD. Additional file 6 shows the calculated effect sizes on the anxiety-related outcomes for workers with OCD and PTSD, respectively, from the studies that were included in the different comparisons. Figure 3 shows a calculated pooled effect-size of a meta-analysis of two studies concerning anxiety-related outcomes of OCD. The data used to calculate the SMDs of the two experimental groups in Aigner et al. were kindly provided upon our request [37]. Data from Marks et al. and Cobb et al. were not requested as these studies were conducted over twenty years ago [43,40].

#### Work-related and anxiety-related outcomes for workers with OCD

##### Exposure in vivo compared with SSRIs

We found significant positive work-related effects for exposure in vivo as part of CBT applied in a group compared with medication treatment (SSRIs), and for exposure in vivo applied along with medication (SSRIs) compared with medication alone (Additional file 5, studies 1a,1b) [37]. The calculated SMDs with 95% confidence intervals were 1.02 (0.48, 1.55) and 0.73 (0.25, 1.20), respectively. These effect sizes are considered to be large (>0.8) and medium-sized (>0.2), respectively. The quality of evidence for these effects was low. We also found a significant positive anxiety-related effect of exposure in vivo in both comparisons (Additional file 6, studies 1a,1b) [37]. The calculated SMDs with 95% confidence intervals were 0.87 (0.34, 1.39) and 1.00 (0.52, 1.49), respectively, which are large effect sizes (>0.8). The quality of evidence for these effects was low.

##### Exposure in vivo compared with self-relaxation

We found a significant positive work-related effect for exposure in vivo as part of CBT applied via a computer compared with systematic self-relaxation and applied by a clinician compared with systematic self-relaxation (Additional file 5, studies 2a,2b) [39]. The calculated SMDs with 95% confidence intervals were 0.35 (0.08, 0.79) and 0.72 (0.28, 1.17), respectively. These effect sizes are both considered medium-sized (>0.2). The quality of evidence for these work-related effects was judged moderate. We also found a significant positive anxiety-related effect of exposure in vivo in both comparisons (Additional file 6, studies 2a,2b) [39]. The calculated SMDs with 95% confidence intervals were 0.72 (0.28, 1.17) and 1.01 (0.55, 1.47), respectively, which are medium-sized (>0.2) and large effects (>0.8). The quality of evidence for these anxiety-related effects was moderate.

##### Exposure in vivo compared with anti-exposure

We found a significant positive work-related effect of exposure in vivo combined with medication (clomipramine) compared with medication combined with anti-exposure work at home (Additional file 5, study 4) [43]. Anti-exposure means avoiding anxiety or ritual-evoking stimuli as much as possible. However, due to a lack of raw data, a SMD could not be calculated. The quality of evidence for this work-related effect was high. The anxiety-related effects in the described comparison were also significantly positive on 9 out of 14 outcome measures (Additional file 6, study 4) [43]. No significant effect was reported in the other 5 of 14 measures. We concluded that overall there was a significant positive anxiety-related effect. Due to a lack of raw data, SMDs for anxiety-related outcomes were not calculated. The quality of evidence for this anxiety-related effect was judged high.

##### Exposure in vivo compared with response prevention

In one study we found a non-significant work-related effect of exposure in vivo applied at home compared with response prevention and exposure in vivo applied at home plus response prevention compared with response prevention alone (Additional file 5, studies 3a,3b) [41]. The calculated SMDs with 95% confidence intervals were 0.12 (-1.02, 1.27) and 0.68 (-0.43, 1.79), respectively. These effect sizes are considered small (<0.2) and medium-sized (>0.2). The quality of evidence for these work-related effects was moderate. The anxiety-related effects of exposure in vivo in both comparisons were also non-significant (Additional file 6, studies 3a,3b) [41]. The calculated SMDs with 95% confidence intervals on (self-rated) obsessions were -0.52 (-1.69, 0.64) and -0.03 (-1.10, 1.04), respectively. The calculated SMDs with 95% confidence intervals on compulsions were 0.15 (-0.99, 1.30) and 0.21 (-0.86, 1.29), respectively. These effect sizes are considered small (<0.2) and medium-sized (>0.2). The quality of evidence for these anxiety-related effects was judged moderate.

##### Exposure in vivo compared with marital therapy

One study demonstrated a non-significant work-related effect of exposure in vivo compared with marital therapy for a mixed group of workers with OCD and severe phobia (Additional file 5, study 5) [40]. A SMD could not be calculated, due to a lack of data. The quality of evidence for the reported effect was judged moderate. Except for one positive significant anxiety-related outcome (i.e., two main phobic-obsessive target problems), we found non-significant effects on three other anxiety-related outcome measures (Additional file 6, study 5) [40]. We could not calculate a SMD for these outcomes due to a lack of data. The quality of evidence for the anxiety-related effects was moderate.

##### Meta-analysis OCD

We could not perform a meta-analysis on the work-related and anxiety-related data of the five OCD studies due to clinical heterogeneity, particularly in the control groups (i.e., some received SSRI medication, others received systematic self-relaxation or response prevention therapies) [37,39-41,43].

However, we did perform a meta-analysis of the outcomes of two studies that represents the net contribution of exposure in vivo. The comparison between exposure in vivo as part of CBT applied in a group plus medication (SSRIs) compared with medication (SSRIs) alone and the comparison between exposure in vivo applied at home plus response prevention compared with response prevention only (Additional file 5 and 6, studies 1b,3b) [37,41] were included in this meta-analysis. Within both comparisons, exposure in vivo was the only difference between groups. We present the calculated pooled effect size of the work-related single effect by means of a meta-analysis in Figure 2. The pooled SMD with 95% confidence interval was 0.72 (0.28, 1.15), which favours treatment programmes with exposure in vivo treatment over treatment programmes without exposure in vivo. The pooled effect size is considered medium sized (>0.2). I^2 ^was 0%, indicating no statistical heterogeneity. The quality of evidence for the pooled effect was judged moderate.

We present the calculated pooled effect size of the anxiety-related single effect of exposure in vivo by means of a meta-analysis in Figure 3. We found a pooled SMD with 95% confidence interval of 0.54 (-0.16, 1.24), which is a non-significant effect. We used a random-effects model, as I^2 ^was greater than 50%. The quality of evidence for this pooled effect was judged moderate.

#### Work-related and anxiety related outcomes for workers with PTSD

##### Exposure in vivo compared with waiting list

We found a significant positive work-related effect for prolonged exposure in vivo compared with a waiting list condition and prolonged exposure in vivo plus cognitive restructuring compared with a waiting list condition (Additional file 5, studies 6a, 6b) [42]. The calculated SMDs with 95% confidence intervals were 0.82 (0.12, 1.52) and 0.77 (0.02, 1.51), respectively. These effect sizes are considered large (>0.8) and medium-sized (>0.2). The quality of evidence for these effects was judged high.

Additional file 6 also shows the calculated effect sizes of exposure in vivo on the PTSD symptoms of workers with PTSD. The calculated SMDs of three other anxiety-related outcome measures are available on request. We found a significant positive effect of prolonged exposure on PTSD symptoms in both comparisons (Additional file 6, studies 6a, 6b) [42]. The calculated SMDs with 95% confidence intervals were 1.92 (1.35, 2.49) and 1.80 (1.22, 2.38), respectively, both large effect sizes (>0.8). The quality of evidence for these anxiety-related effects was judged high.

##### Exposure in vivo compared with imaginal exposure

We found a non-significant work-related effect of exposure in vivo compared with imaginal exposure (Additional file 5, study 7) [38]. The calculated Odds Ratio with 95% confidence interval was 1.27 (0.49, 3.31). The quality of evidence for this work-related effect was judged low. We also found a non-significant anxiety-related effect of exposure in vivo compared with imaginal exposure (Additional file 6, study 7) [38]. The calculated SMD with 95% confidence interval was -0.11 (-0.55, 0.33), which is considered a small effect size (effect size <0.2). The quality of evidence for this anxiety-related effect was judged low.

##### PTSD meta-analysis

We did not perform a meta-analysis of the two PTSD studies due to heterogeneity in the control groups (waiting list and imaginal exposure) and included workers (PTSD and PTSD with non-visual flashbacks). Furthermore, we did not perform a meta-analysis of the net contribution of exposure in vivo as we could not find PTSD studies in which exposure in vivo was the only difference between groups.

## Discussion

We included seven studies in which workers were treated with exposure in vivo as the major component of anxiety treatment. Two studies had a high quality of evidence, three studies had a moderate quality of evidence, and two studies had a low quality of evidence. Four studies concerned workers with obsessive-compulsive disorders (OCD), one study concerned a mixed group of workers with OCD or severe phobias, and two studies concerned workers with post-traumatic disorder (PTSD). For OCD we found a low to high quality of evidence that exposure in vivo can reduce adverse work-related outcomes with a medium to large effect in five different modalities and comparisons (Group CBT vs. SSRIs, group CBT plus SSRIs vs. SSRIs, clinician guided CBT vs. systematic self-relaxation, exposure homework combined with Clomipramine vs. Clomipramine with anti-exposure homework). We found moderate evidence that exposure in vivo did not reduce adverse work-related outcomes in workers with OCD in three other modalities and comparisons (computer CBT at home via telephone vs. systematic self-relaxation, exposure at home vs. response prevention, exposure at home plus response prevention vs. response prevention). Moreover, in a meta-analysis of two OCD studies representing the net contribution of exposure in vivo, we found moderate evidence of a medium-sized effect on work-related outcome. Furthermore, we found that this work-related effect was combined with moderate evidence of no increase in anxiety related outcomes. Based on both meta-analyses, we may conclude that there is moderate evidence that anxiety treatments including exposure in vivo can reduce adverse work-related outcomes in workers with OCD with a medium-sized effect, and do not increase anxiety. For workers with PTSD, we found a high quality of evidence that exposure in vivo can reduce adverse work-related outcomes with a medium to large effect in two different modalities and comparisons (prolonged exposure vs. waiting list, prolonged exposure plus cognitive restructuring vs. waiting list). We found a low quality of evidence that exposure in vivo compared with imaginal exposure did not differ in improving work-related outcomes. The work-related effects for workers with PTSD were obtained without increasing anxiety.

That we found only seven relevant studies for this review after a comprehensive and sensitive literature search is a remarkable finding in and of itself. We included four studies involving workers with OCD, one study involving a mixed group of workers with OCD and severe phobias, two studies involving workers with PTSD, and no studies involving workers with other anxiety disorders. These findings are in sharp contrast with the prevalence of studies that have reported an association between a variety of anxiety disorders and work-related outcomes such as absence due to sickness, presenteeism, and decreased productivity. Our findings in workers with OCD are consistent with the low number of studies with work-related results in Steketee's review focussed on OCD and social functioning, which is a broader concept and includes work-related, social, and leisure-related outcomes [14]. Our review had three studies in common with Steketee's review.

Another suprising finding is that only one of the seven included studies investigated an outcome parameter related to return-to-work, i.e., employment status while increased sickness absence is frequently reported in cross-sectional studies [7,10,11,38]. The other studies investigated self-reported work functioning, which is a broader concept than return-to-work. In future studies, return-to-work outcomes should be evaluated more often.

A third noteworthy finding is that no controlled studies have been performed in which exposure in vivo was aimed at specific anxiety-provoking work situations, i.e., situations related to specific tasks, social relations, or workplaces. Avoidance of such tasks, relationships, or workplaces can hinder good job performance. Specific work-related anxiety complaints and anxiety disorders such as work-related panic, work-related phobia, work-related social phobia, and work-related generalised anxiety exist as clinical phenomena partly independent of anxiety disorders in general and therefore deserve specific therapeutic attention [44].

A strength of this review is its sensitive and comprehensive search of four electronic databases, using search words partly based on prior bibliographic research. Furthermore, we used the GRADE criteria, recommended by the Cochrane Collaboration, to judge the quality of evidence of included studies.

Although the distinction between high and low-risk of bias or high and low quality of evidence of studies is still controversial, it offers the advantage that the process of labelling the risk of bias and the quality of evidence is explicit and transparent [45].

Usually, blinding of participants and health care providers is one of the criteria for assessing risk-of-bias. We excluded these aspects of the assessment as they lack applicability in this type of intervention study. Workers cannot be blinded adequately to the intervention they receive and health care providers cannot be blinded sufficiently to the intervention they provide [34,35]. Blinding of assessors is the only criterion that remains to reduce the risk of bias in this type of study.

A weak point of this review is that it is not possible to evaluate the compliance with exposure in vivo because this was not reported in the included studies. Thus, we are uncertain about the minimal dose of exposure in vivo that could have reduced work-related adverse outcome. Furthermore, all the included studies evaluated exposure in vivo in the context of a much broader treatment strategy, rather than by itself. This could have diminished or strengthened the effect of the treatment, as interaction may have occurred between the effects of exposure in vivo and the effects of other components of the intervention strategy.

A methodological consideration of this review is that we calculated SMDs based on the final post-treatment scores, not on the change score. According to the Cochrane Handbook for Systematic Reviews of Interventions, this is an adequate option [36]. We chose to do so because final post-treatment scores were available in five of the seven included studies, so we could compare SMDs between individual studies. In contrast, the change score was only available in one study [39].

Generalising the results of this review to workers with OCD must be done cautiously, as we found mixed results in different comparisons between groups. The results of this review cannot be generalised to anxiety disorders other than OCD without discussion, as our included studies mainly concerned workers with OCD and we found mixed results of exposure in vivo in two studies that included workers with PTSD.

In future research, priority should be given to high-quality randomised controlled trials (RCTs) applying exposure in vivo to a variety of anxiety disorders, and measuring work-related outcomes as well as anxiety symptoms. In particular, work-related outcomes such as work functioning, productivity, and absence due to sickness should be evaluated. Future research should be aimed at work-related anxiety complaints and disorders, as they can be distinguished from anxiety disorders in general. Reliable and valid work-related outcome measures are needed to evaluate interventions for general anxiety and for work-related anxiety. Recently developed measures, such as the MINI work anxiety interview and the Occupational Functioning Scale, can be considered for use in such research [46]. Exposure in vivo should be compared with other effective treatments for anxiety disorders that are part of usual care such as SSRIs, as these are more stringent control conditions than waiting list or relaxation.

## Conclusions

Exposure-in-vivo containing interventions can improve work functioning in workers with OCD and PTSD better compared to various other anxiety treatments or a waiting-list. We recommend that it should be studied how the results of these studies can be transferred to the practice of occupational health professionals and how clinicians can make better use of them to improve work-related outcomes.

## Competing interests

The authors declare that they have no competing interests.

## Authors' contributions

EK, EN, and KN developed the search strategy and participated in the inclusion and exclusion of articles, quality assessment, and data analysis. KN, JvdK, and FvD participated in the design and co-ordination of the review. EN and EK wrote the manuscript. All authors provided comments on the drafts of the manuscript and have read and approved the final version.

## Pre-publication history

The pre-publication history for this paper can be accessed here:

http://www.biomedcentral.com/1471-2458/10/598/prepub

## Supplementary Material

Additional file 1 **Searchstrings**. The words used to search the databases PsycINFO, Cinahl, Embase, and Medline, are presented. Click here for file

Additional file 2 **Characteristics of seven included studies**. For each study, the study number, comparison a/b, and reference, first author, quality of evidence rating according to GRADE-criteria, design, participants (number, type of anxiety disorder, age, % working at baseline), country and setting of study, follow-up period, the intervention programme with exposure in vivo (E), the treatment programme of the control group (C), and the work- and anxiety-related outcomes are presented. Click here for file

Additional file 3 **For seven included studies containing 11 comparisons the study number, comparison a/b, and reference, components, duration, and provider of the treatment programme of the experimental group with exposure in vivo, and that of the treatment programme of the control group without exposure in vivo, are presented**. Click here for file

Additional file 4 **For seven included studies and one meta-analysis the judgement of quality of evidence based on five GRADE criteria**. For each study the study number, comparison a/b, and reference, first author, publication year, design (RCT/CT), and the concluding judgement of the quality of evidence based on the following five GRADE criteria: 1. conclusion of risk of bias transformed in GRADE-assessment of study limitation, 2. indirectness of evidence, 3. unexplained heterogeneity or inconsistency of results, 4. imprecision of results, 5. high probability of publication bias, are presented. Click here for file

Additional file 5 **For seven included studies containing 11 comparisons and one meta-analysis the effects on the work-related outcomes of workers with OCD and PTSD**. For each comparison the study number, comparison a/b, and reference, quality of evidence, anxiety disorder, experimental group with exposure in vivo, control group without exposure in vivo, the effect-size of the work-related effect in SMD or OR, its 95% confidence interval, the reported test statistics, and p-value, are presented, as far as data were available. Click here for file

Additional file 6 **For seven included studies containing 11 comparisons and one meta-analysis the effects on the anxiety-related outcomes of workers with OCD and PTSD, are presented**. For each comparison the study number, comparison a/b, and reference, quality of evidence, anxiety disorder, experimental group with exposure in vivo, control group without exposure in vivo, the effect-size of the anxiety-related effect in SMD or OR, its 95% confidence interval, the reported test statistics, and p-value, are presented, as far as data were available. Click here for file
