# Supplementary material for: Exposure-in-vivo containing interventions to improve work functioning of workers with anxiety disorder: a systematic review
Source: BMC Public Health. 2010 Oct 11;10:598. doi: 10.1186/1471-2458-10-598 (PMC3224747; doi:10.1186/1471-2458-10-598)
Supplement: Additional file 1 — Searchstrings. The words used to search the databases PsycINFO, Cinahl, Embase, and Medline, are presented. [file 1471-2458-10-598-S1.DOC]

PsycINFO (Silverplatter)

| #84 | #83 and #62 and #27 and #10 and (PY:PSYI = 1972-2007) |
| --- | --- |
| #83 | (fear*) or (fright*) or (irratio*) or (preoccupation) or (ruminat*) or (compuls*) or (catas*) or (escap*) or (avoid*) or (anx*) or (afraid*) or (angst*) or (agoraphobia) or (GAD) or (OCD) or (Phobia) or (PTSD) or (worr*) or (panic*) or (obses*) or (intrusive) |
| #62 | (vocational rehabilitation and (aj=yes)) or (employment and (aj=yes)) or (supported employment and (aj=yes)) or (occupational intervention* and (aj=yes)) or (occupational therap* and (aj=yes)) or (return to work and (aj=yes)) or (explode Work-Related-Illnesses and (aj=yes)) or (explode Reemployment and (aj=yes)) or (Occupational-Therapy and (aj=yes)) or (Occupational-Stress and (aj=yes)) or (explode Occupational-Status and (aj=yes)) or (explode Employee-Absenteeism and (aj=yes)) or (explode Disability-Management and (aj=yes)) or (explode Vocational-Rehabilitation and (aj=yes)) or (explode Occupational-Guidance and (aj=yes)) or (explode Job-Satisfaction and (aj=yes)) or (vocational and (aj=yes)) or (explode Employee-Leave-Benefits and (aj=yes)) or (explode Employability and (aj=yes)) or (explode Disability-Evaluation and (aj=yes)) or (job and (aj=yes)) or (occupation* and (aj=yes)) or (disability pension and (aj=yes)) or (retirement and (aj=yes)) or (sick* absence and (aj=yes)) or (sick leave and (aj=yes)) or (unemployment and (aj=yes)) or (employed and (aj=yes)) or (unemployed and (aj=yes)) or (occupational health and (aj=yes)) or (occupational health services and (aj=yes)) or (absenteeism and (aj=yes)) or (vocational guidance and (aj=yes)) or (work capacity evaluation and (aj=yes)) |
| #27 | #26 not #25 |
| #26 | (crossover) or (placebo*) or ((singl* or doubl* or trebl* or tripl*) near25 (blind* or dummy or mask*)) or (random*) or (explode mental health program evaluation) or (explode treatment effectiveness evaluation) or (explode experimental design) or (explode placebo) or ((clin* or control* or compare* or evaluat* or prospective*) near25 (trial* or studi* or study)) or (allocat*) or (assign*) |
| #25 | #22 not #24 |
| #24 | ((human or inpatient or outpatient) in po) and (animal in po) |
| #22 | animal in po |
| #10 | (extinc*) or (reinforc*) or ("in vivo*") or (stimulus*) or (skinner) or (habitua*) or (exposure*) or (behavio*) or (conditioning*) |

Cinahl

1. exp job performance/

2. exp job re-entry/

3. exp employment/

4. exp occupational health/

5. exp rehabilitation, vocational/

6. exp sick leave/

7. exp work/

8. exp disability evaluation/

9. exp Occupational Therapy/

10. return to work.mp.

11. occupational therap$.mp.

12. occupational intervention$.mp.

13. supported employment.mp.

14. employment.mp.

15. vocational rehabilitation.mp.

16. work capacity evaluation.mp.

17. vocational guidance.mp.

18. absenteeism.mp.

19. occupational health services.mp.

20. occupational health.mp.

21. unemployed.mp.

22. employed.mp.

23. unemployment.mp.

24. sick leave.mp.

25. sick$ absence.mp.

26. retirement.mp.

27. disability pension.mp.

28. occupation$.mp.

29. job.mp.

30. vocational.mp.

31. 1 or 2 or 3 or 4 or 5 or 6 or 7 or 8 or 9 or 10 or 11 or 12 or 13 or 14 or 15 or 16 or 17 or 18 or 19 or 20 or 21 or 22 or 23 or 24 or 25 or 26 or 27 or 28 or 29 or 30

32. clinical trial.pt.

33. exp Clinical trials/

34. (clin$ adj25 trial$).ti,ab.

35. placebos.sh.

36. placebo$.ti,ab.

37. random$.ti,ab.

38. exp evaluation studies/

39. prospective studies.sh.

40. (control$ or prospectiv$ or volunteer$).ti,ab.

41. ((singl$ or doubl$ or tripl$ or trebl$) adj25 (blind$ or mask$ or dummy$)).mp.

42. 32 or 33 or 34 or 35 or 36 or 37 or 38 or 39 or 40 or 41

43. fear$.mp. [mp=title, subject heading word, abstract, instrumentation]

44. fright$.mp. [mp=title, subject heading word, abstract, instrumentation]

45. afraid$.mp. [mp=title, subject heading word, abstract, instrumentation]

46. angst$.mp. [mp=title, subject heading word, abstract, instrumentation]

47. agoraphobia.mp. [mp=title, subject heading word, abstract, instrumentation]

48. GAD.mp. [mp=title, subject heading word, abstract, instrumentation]

49. OCD.mp. [mp=title, subject heading word, abstract, instrumentation]

50. PTSD.mp. [mp=title, subject heading word, abstract, instrumentation]

51. Phobia.mp. [mp=title, subject heading word, abstract, instrumentation]

52. worr$.mp. [mp=title, subject heading word, abstract, instrumentation]

53. panic$.mp. [mp=title, subject heading word, abstract, instrumentation]

54. obses$.mp. [mp=title, subject heading word, abstract, instrumentation]

55. intrusive$.mp. [mp=title, subject heading word, abstract, instrumentation]

56. irratio$.mp. [mp=title, subject heading word, abstract, instrumentation]

57. preoccupation.mp. [mp=title, subject heading word, abstract, instrumentation]

58. ruminat$.mp. [mp=title, subject heading word, abstract, instrumentation]

59. compuls$.mp. [mp=title, subject heading word, abstract, instrumentation]

60. catas$.mp. [mp=title, subject heading word, abstract, instrumentation]

61. escap$.mp. [mp=title, subject heading word, abstract, instrumentation]

62. avoid$.mp. [mp=title, subject heading word, abstract, instrumentation]

63. anx$.mp. [mp=title, subject heading word, abstract, instrumentation]

64. 43 or 44 or 45 or 46 or 47 or 48 or 49 or 50 or 51 or 52 or 53 or 54 or 55 or 56 or 57 or 58 or 59 or 60 or 61 or 62 or 63

65. vivo$.mp. [mp=title, subject heading word, abstract, instrumentation]

66. reinf$.mp. [mp=title, subject heading word, abstract, instrumentation]

67. habitu$.mp. [mp=title, subject heading word, abstract, instrumentation]

68. exti$.mp. [mp=title, subject heading word, abstract, instrumentation]

69. conditioni$.mp. [mp=title, subject heading word, abstract, instrumentation]

70. Skinner.mp. [mp=title, subject heading word, abstract, instrumentation]

71. behav$.mp.

72. stimulus$.mp.

73. expos$.mp. [mp=title, subject heading word, abstract, instrumentation]

74. 65 or 66 or 67 or 68 or 69 or 70 or 71 or 72 or 73

75. 31 and 42 and 64 and 74

76. limit 75 to yr="1982 - 2007"

Embase

1. fear$.mp. [mp=title, abstract, subject headings, heading word, drug trade name, original title, device manufacturer, drug manufacturer name]

2. fright$.mp. [mp=title, abstract, subject headings, heading word, drug trade name, original title, device manufacturer, drug manufacturer name]

3. afraid$.mp. [mp=title, abstract, subject headings, heading word, drug trade name, original title, device manufacturer, drug manufacturer name]

4. angst$.mp. [mp=title, abstract, subject headings, heading word, drug trade name, original title, device manufacturer, drug manufacturer name]

5. agoraphobia.mp. [mp=title, abstract, subject headings, heading word, drug trade name, original title, device manufacturer, drug manufacturer name]

6. GAD.mp. [mp=title, abstract, subject headings, heading word, drug trade name, original title, device manufacturer, drug manufacturer name]

7. OCD.mp. [mp=title, abstract, subject headings, heading word, drug trade name, original title, device manufacturer, drug manufacturer name]

8. Phobia.mp. [mp=title, abstract, subject headings, heading word, drug trade name, original title, device manufacturer, drug manufacturer name]

9. PTSD.mp. [mp=title, abstract, subject headings, heading word, drug trade name, original title, device manufacturer, drug manufacturer name]

10. worr$.mp. [mp=title, abstract, subject headings, heading word, drug trade name, original title, device manufacturer, drug manufacturer name]

11. panic$.mp. [mp=title, abstract, subject headings, heading word, drug trade name, original title, device manufacturer, drug manufacturer name]

12. obses$.mp. [mp=title, abstract, subject headings, heading word, drug trade name, original title, device manufacturer, drug manufacturer name]

13. intrusive$.mp. [mp=title, abstract, subject headings, heading word, drug trade name, original title, device manufacturer, drug manufacturer name]

14. irratio$.mp. [mp=title, abstract, subject headings, heading word, drug trade name, original title, device manufacturer, drug manufacturer name]

15. preoccupation.mp. [mp=title, abstract, subject headings, heading word, drug trade name, original title, device manufacturer, drug manufacturer name]

16. ruminat$.mp. [mp=title, abstract, subject headings, heading word, drug trade name, original title, device manufacturer, drug manufacturer name]

17. compuls$.mp. [mp=title, abstract, subject headings, heading word, drug trade name, original title, device manufacturer, drug manufacturer name]

18. catas$.mp. [mp=title, abstract, subject headings, heading word, drug trade name, original title, device manufacturer, drug manufacturer name]

19. escap$.mp. [mp=title, abstract, subject headings, heading word, drug trade name, original title, device manufacturer, drug manufacturer name]

20. avoid$.mp. [mp=title, abstract, subject headings, heading word, drug trade name, original title, device manufacturer, drug manufacturer name]

21. anx$.mp. [mp=title, abstract, subject headings, heading word, drug trade name, original title, device manufacturer, drug manufacturer name]

22. 1 or 2 or 3 or 4 or 5 or 6 or 7 or 8 or 9 or 10 or 11 or 12 or 13 or 14 or 15 or 16 or 17 or 18 or 19 or 20 or 21

23. Skinner.mp. [mp=title, abstract, subject headings, heading word, drug trade name, original title, device manufacturer, drug manufacturer name]

24. stimulus$.mp.

25. in vivo$.mp.

26. reinforc$.mp.

27. extinc$.mp.

28. conditioning$.mp.

29. behavio$.mp.

30. exposure$.mp.

31. habitua$.mp.

32. 23 or 24 or 25 or 26 or 27 or 28 or 29 or 30 or 31

33. occupational therapy.mp.

34. occupational disease.mp.

35. occupational medicine.mp.

36. employment.mp.

37. vocational rehabilitation.mp.

38. work capacity.mp.

39. vocational guidance.mp.

40. absenteeism.mp.

41. occupational health service.mp.

42. occupational health.mp.

43. unemployment.mp.

44. retirement.mp.

45. occupation.mp.

46. vocation.mp.

47. disability evaluation.mp.

48. return to work.mp.

49. occupational intervention$.mp.

50. supported employment.mp.

51. unemployed.mp.

52. employed.mp.

53. sick leave.mp.

54. sick$ absence.mp.

55. disability pension.mp.

56. job.mp.

57. vocational.mp.

58. exp work/

59. (disability adj (work or occupation$ or vocation$ or job)).mp.

60. 33 or 34 or 35 or 36 or 37 or 38 or 39 or 40 or 41 or 42 or 43 or 44 or 45 or 46 or 47 or 48 or 49 or 50 or 51 or 52 or 53 or 54 or 55 or 56 or 57 or 58 or 59

61. controlled study.de.

62. clinical trial.de.

63. major clinical study.de.

64. randomized controlled trial.de.

65. double blind procedure.de.

66. clinical article.de.

67. random$.mp.

68. compar$.mp.

69. control$.mp.

70. follow up$.mp.

71. ((singl$ or doubl$ or tripl$ or trebl$) adj (blind$ or mask$ or dummy)).mp.

72. placebo$.mp.

73. (clinic$ adj (trial$ or study or studies$)).mp.

74. 61 or 62 or 63 or 64 or 65 or 66 or 67 or 68 or 69 or 70 or 71 or 72 or 73

75. human.de.

76. nonhuman.de.

77. 75 and 76

78. 76 not 77

79. 74 not 78

80. 22 and 32 and 60 and 79

80. limit 80 to yr="1980 - 2007"

Medline

1. fear$.mp. [mp=ti, ot, ab, nm, hw]

2. fright$.mp. [mp=ti, ot, ab, nm, hw]

3. afraid$.mp. [mp=ti, ot, ab, nm, hw]

4. angst$.mp. [mp=ti, ot, ab, nm, hw]

5. agoraphobia.mp. [mp=ti, ot, ab, nm, hw]

6. GAD.mp. [mp=ti, ot, ab, nm, hw]

7. OCD.mp. [mp=ti, ot, ab, nm, hw]

8. Phobia.mp. [mp=ti, ot, ab, nm, hw]

9. PTSD.mp. [mp=ti, ot, ab, nm, hw]

10. worr$.mp. [mp=ti, ot, ab, nm, hw]

11. panic$.mp. [mp=ti, ot, ab, nm, hw]

12. obses$.mp. [mp=ti, ot, ab, nm, hw]

13. intrusive$.mp. [mp=ti, ot, ab, nm, hw]

14. irratio$.mp. [mp=ti, ot, ab, nm, hw]

15. preoccupation.mp. [mp=ti, ot, ab, nm, hw]

16. ruminat$.mp. [mp=ti, ot, ab, nm, hw]

17. compuls$.mp. [mp=ti, ot, ab, nm, hw]

18. catas$.mp. [mp=ti, ot, ab, nm, hw]

19. escap$.mp. [mp=ti, ot, ab, nm, hw]

20. avoid$.mp. [mp=ti, ot, ab, nm, hw]

21. anx$.mp. [mp=ti, ot, ab, nm, hw]

22. 1 or 2 or 3 or 4 or 5 or 6 or 7 or 8 or 9 or 10 or 11 or 12 or 13 or 14 or 15 or 16 or 17 or 18 or 19 or 20 or 21

23. Skinner.mp. [mp=ti, ot, ab, nm, hw]

24. stimulus$.mp.

25. in vivo$.mp.

26. reinforc$.mp.

27. extinc$.mp.

28. conditioning$.mp.

29. behavio$.mp.

30. exposure$.mp.

31. habitua$.mp.

32. 23 or 24 or 25 or 26 or 27 or 28 or 29 or 30 or 31

33. exp Occupational Therapy/

34. exp Occupational Diseases/

35. exp Occupational Medicine/

36. exp Disability Evaluation/

37. exp WORK/

38. return to work.mp.

39. occupational therap$.mp.

40. occupational intervention$.mp.

41. supported employment.mp.

42. employment.mp.

43. vocational rehabilitation.mp.

44. work capacity evaluation.mp.

45. vocational guidance.mp.

46. absenteeism.mp.

47. occupational health services.mp.

48. occupational health.mp.

49. unemployed.mp.

50. employed.mp.

51. unemployment.mp.

52. sick leave.mp.

53. sick$ absence.mp.

54. retirement.mp.

55. disability pension.mp.

56. occupation$.mp.

57. job.mp.

58. vocational.mp.

59. 33 or 34 or 35 or 36 or 37 or 38 or 39 or 40 or 41 or 42 or 43 or 44 or 45 or 46 or 47 or 48 or 49 or 50 or 51 or 52 or 53 or 54 or 55 or 56 or 57 or 58

60. randomized-controlled-trial.pt.

61. controlled clinical trial.pt.

62. randomized controlled trials.sh.

63. random allocation.sh.

64. double blind method.sh.

65. single blind method.sh.

66. clinical trial.pt.

67. exp Clinical trials/

68. (clin$ adj25 trial$).ti,ab.

69. ((singl$ or doubl$ or tripl$ or trebl$) adj25 (blind$ or mask$ or dummy$)).mp.

70. placebos.sh.

71. placebo$.ti,ab.

72. random$.ti,ab.

73. research design.sh.

74. comparative study.sh.

75. exp evaluation studies/

76. follow up studies.sh.

77. prospective studies.sh.

78. (control$ or prospectiv$ or volunteer$).ti,ab.

79. exp Treatment Outcome/

80. 60 or 61 or 62 or 63 or 64 or 65 or 66 or 67 or 68 or 69 or 70 or 71 or 72 or 73 or 74 or 75 or 76 or 77 or 78 or 79

81. (ANIMALS not HUMAN).sh.

82. 80 not 81

83. 22 and 32 and 59 and 82

84. limit 83 to yr="1966 - 2007"
