# Supplementary material for: Exposure-in-vivo containing interventions to improve work functioning of workers with anxiety disorder: a systematic review
Source: BMC Public Health. 2010 Oct 11;10:598. doi: 10.1186/1471-2458-10-598 (PMC3224747; doi:10.1186/1471-2458-10-598)
Supplement: Additional file 2 — Characteristics of seven included studies. For each study, the study number, comparison a/b, and reference, first author, quality of evidence rating according to GRADE-criteria, design, participants (number, type of anxiety disorder, age, % working at baseline), country and setting of study, follow-up period, the intervention programme with exposure in vivo (E), the treatment programme of the control group (C), and the work- and anxiety-related outcomes are presented. [file 1471-2458-10-598-S2.PDF]

**Study number, comparison a/b, and reference**	**First author and publication year Design (RCT/CT)**	**Participants: Total number (N) Type of anxiety disorder (mean chronicity in mnth/yrs) Age range % working at baseline Country, setting Follow-up period**	**Experimental condition (E): Intervention programme *with* exposure in vivo (number of completers)**	**Control condition (C): Programme *without* exposure in vivo (C) (number of completers)**	**Work-related outcome**	**Anxiety-related outcome**
1a [37]	**Aigner (2004)**<br>CT	N=155<br>OCD (17.4 yrs)<br>18–60 yrs<br>39% working<br>Austria, University Clinic<br>10 sessions (CBT) or 12 wks (medication)	Group CBT<br>(n=35)	Medication (SSRIs; n=27)	Sheehan Disability Scale (among others work; assessor-rated)	Obsessions and compulsions<br>(Yale-Brown Obsessive Compulsive Scale, Y-BOCS, 1991)
1b [37]	**Aigner (2004)**<br>CT	N=155<br>OCD (17.4 yrs)<br>18–60 yrs<br>39% working<br>Austria, University Clinic<br>10 sessions (CBT) or 12 wks (medication)	Group CBT + medication<br>(n=53)	Medication (SSRIs; n=28)	Sheehan Disability Scale (among others work; assessor-rated)	Obsessions and compulsions<br>(Yale-Brown Obsessive Compulsive Scale, Y-BOCS, 1991)
2a [39]	**Greist (2002)**<br>RCT	N=218<br>OCD (22 yrs)	Computer CBT (home via telephone; n=55)	Systematic self-relaxation<br>(n=33)	Work and Social Adjustment Scale	Obsessions and compulsions<br>(Yale-Brown Obsessive Compulsive Scale, Y-

		15–80 yrs<br>% working not reported<br>USA, 8 outpatient practice sites, and at home<br>26 wks			(among others work; self-rated)	BOCS, 1991)
2b [39]	**Greist (2002)**<br>RCT	N=218<br>OCD (22 yrs)<br>15–80 yrs<br>% working not reported<br>USA, 8 outpatient practice sites, and at home<br>26 wks	Clinician CBT private practices (n=55)	Systematic self-relaxation (n=33)	Work and Social Adjustment Scale (among others work; self-rated)	Obsessions and compulsions<br>(Yale-Brown Obsessive Compulsive Scale, Y-BOCS, 1991)
3a [41]	**Foa (1984)**<br>CT	N=32<br>OCD (9.8 yrs)<br>19–54 yrs<br>% working not reported<br>USA, Temple University<br>12 months (range 3–24 months)	Exposure at home (n=11; 3–18 months)	Response prevention (n=4)	General Functioning: a.o. work scale (0–8: 0=no interference, 8=severe interference; assessor-rated)	13 obsessive and compulsive measures<br>2 anxiety measures
3b [41]	**Foa (1984)**<br>CT	N=32<br>OCD (9.8 yrs)<br>19–54 yrs<br>% working not reported<br>USA, Temple University<br>12 months (range 3–24 months)	Exposure at home + response prevention (n=10; 7-24 months)	Response prevention (n=5)	General Functioning: a.o. work scale (0–8: 0=no interference, 8=severe interference; assessor-rated)	13 obsessive and compulsive measures<br>2 anxiety measures

4 [43]	**Marks (1988)**<br>RCT	N=24<br>OCD (12 yrs)<br>18–60 yrs<br>% working not reported<br>UK, outpatient and inpatient hospital departments<br>17 wks	Clomipramine with exposure homework (n=12)	Clomipramine with anti-exposure homework (n=12)	Work and Social Adjustment Scale (assessor- and self-rated; 0–8 scale; among others work)	14 obsessive and compulsive measures
5 [40]	**Cobb (1980)**<br>RCT	N=11<br>OCD (4×)/severe phobias (7×) (8 yrs)<br>32 yrs (mean)<br>% working not reported<br>UK, setting not reported<br>10 wks	Exposure therapy (n=6)	Marital therapy (n=5)	Maudsley Marital Questionnaire: 5 of 20 items concerned work and social activities (0–8 scale; self- and spouse-rated)	Phobias and obsessions (self- and assessor-rated): two main phobic–obsessive target problems (scale 0–8)<br>Fear Survey Schedule (scale 0–162)<br>OC patients: two main obsessions (daily time taken up or discomfort; scale 0–8)<br>OC checklist (scale 0–117)
6a [42]	**Foa (2005)**<br>(RCT)	N=171 female<br>PTSD (9 yrs)<br>21-41 yrs<br>USA, academic and community clinic<br>12 months<br>58% working	prolonged exposure (PE; n=48)	Wait-list (n= 10)	Work and Social Adjustment Scale: subscale Work	PTSD Symptom Scale (PSS-I assessor-rated interview and PSS-SR self rated report; scale 0-3)
6b [42]	**Foa (2005)**<br>(RCT)	N=171 female<br>PTSD (9 yrs)<br>21-41 yrs<br>USA, academic and community clinic	PE + cognitive restructuring (n=40)	Wait-list (n= 9)	Work and Social Adjustment Scale: subscale Work	PTSD Symptom Scale (PSS-I assessor-rated interview and PSS-SR self rated report; scale 0-3)

		12 months<br>58% working				
7 [38]	**Salyards (2005)**<br>(CT)	N= 80<br>PTSD with non-visual flashbacks (nr. yrs. not reported)<br>18-61 yrs<br>USA, academic clinic<br>12 months<br>0% working	Exposure in vivo (n=40)	Imaginal exposure (n=40)	Employment status (return to work yes/no) and five other specifications of return-to-work	Frequency of flashbacks (self rated log per week; total number)<br>Subjective Units of distress (SUDS; scale 0-10)<br>PTSD symptoms: Impact of Event Scale (IES), 3)<br>State-Trait Anxiety Inventory (STAI; self-rated; scale 1-4)
