# Supplementary material for: Exposure-in-vivo containing interventions to improve work functioning of workers with anxiety disorder: a systematic review
Source: BMC Public Health. 2010 Oct 11;10:598. doi: 10.1186/1471-2458-10-598 (PMC3224747; doi:10.1186/1471-2458-10-598)
Supplement: Additional file 3 — For seven included studies containing 11 comparisons the study number, comparison a/b, and reference, components, duration, and provider of the treatment programme of the experimental group with exposure in vivo, and that of the treatment programme of the control group without exposure in vivo, are presented. [file 1471-2458-10-598-S3.PDF]

| Study number, comparison a/b, and reference | Experimental group with exposure in vivo | | | Control group without exposure in vivo | | |
|---|---|---|---|---|---|---|
| | **Components** | **Duration** | **Provider** | **Components** | **Duration** | **Provider** |
| 1a [37] | – Group therapy (5×): group contact and cohesion, information & rationale, individual goalsetting, problem orientation, preparation of exposure in vivo, training social interaction skills, self-monitoring; 5–8 persons per group<br>– Execution exposure (4×): example of exposure in vivo, appointments and planning exposure homework, building alternative behaviour, individual problem solving<br>– Last session (1×): relapse prevention, compliance talk, feedback<br>– Extra information groups for patients and family/friends (1 hour per 2 weeks) as co-therapist: information (symptoms, goal, progress), coping with OCD (compliance, support), exchange of experiences, individual problem solving | 10 weekly sessions of 2 hours per session | Not reported | Medical therapy<br>Clinical management<br>SSRI (Seatrain, Fluoxetin or Fluvozamin)<br><br>Information and instruction for family/friends when they attend a monitoring session | Minimum of 12 weeks | Not reported |
| 1b [37] | Combined group + medical therapy | 10 sessions group therapy and medication after 3–4 weeks of group therapy | Not reported | Medical therapy<br>Clinical management<br>SSRI (Seatrain, Fluoxetin or Fluvozamin)<br><br>Information and instruction for family/friends when they attend a monitoring session | Minimum of 12 weeks | Not reported |
| 2a [39] | BT STEPS: 9 steps computer behaviour therapy: self-paced workbook by telephone with interactive voice response (12×)<br>Steps 1–3: Education and assessment<br>Steps 4–9: Daily self-exposure and response prevention | 10 weeks of daily exposure for a minimum of 60 minutes | Computer driven self-exposure | Progressive relaxation | Minimal 60 minutes per day relaxation exercises, and relaxation diaries for 10 weeks | Manual and audiotape |
| 2b [39] | Clinician-guided self-exposure:<br>Negotiate self-exposure in vivo<br>Self-exposure homework | 11 sessions, weekly 1 hour and daily 1 hour self-exposure homework recorded in diary | Clinician with behaviour therapy expertise | Progressive relaxation | Minimal 60 minutes per day relaxation exercises, and relaxation diaries for 10 weeks | Manual and audiotape |
| 3a [41] | Exposure in vivo: each session a new contaminant was added; at 6$^{th}$ day the most feared item; 7–15 sessions exposure to all items | 15 daily, 2 hour sessions in 3 weeks; in 4$^{th}$ week therapist visits 2 days for 4 hours at home | 4 therapists experienced in behaviour therapy | Response prevention of ritualistic cleaning and washing; except 10 min shower every 5$^{th}$ day; avoidance allowed; during sessions: discuss avoidance patterns, unexpected contamination, and urges to wash | 15 daily, 2 hour sessions in 3 weeks; in 4$^{th}$ week therapist visits 2 days for 4 hours at home | 4 therapists experienced in behaviour therapy |

| | | | | | | |
|---|---|---|---|---|---|---|
| 3b [41] | Exposure in vivo + response<br>Prevention | 15 daily, 2 hour sessions in 3 weeks; in 4th week therapist visits 2 days for 4 hours at home | 4 therapists experienced in behaviour therapy | Response prevention of ritualistic cleaning and washing; except 10 min shower every 5th day; avoidance allowed; during sessions: discuss avoidance patterns, unexpected contamination, and urges to wash | 15 daily, 2 hour sessions in 3 weeks; in 4th week therapist visits 2 days for 4 hours at home | 4 therapists experienced in behaviour therapy |
| 4 [43] | Clomipramine with exposure homework:<br>Homework instruction sheet, information about exposure, and response prevention<br>Instruction to find someone to model normal behaviour<br>Homework diary<br>Existing coping stimulation if compatible with treatment | 17 weeks of daily homework for 3 hours | Patient | Clomipramine with anti-exposure homework:<br>asked to avoid anxiety or ritual-evoking stimuli as much as possible<br>If urges to ritualise did appear, patients should feel free to do so | 17 weeks of daily homework for 3 hours | Patient |
| 5 [40] | Exposure in vivo: persuading patient for exposure in vivo, modelling some exposure tasks, supervision of spouse during homework | 10 weeks, weekly 50 minutes | Psychiatrist<br>Spouse as co-therapist | Marital therapy: agreeing contracts and goals, clarifying difficulties, sexual skills training, improving communication | 10 weeks, weekly 50 minutes | Psychiatrist |
| 6a [42] | Prolonged exposure in vivo: treatment rationale, reviewed homework, conducted imaginal exposure to trauma memory for 30–45 min, discussed the imaginal exposure, and assigned in vivo and imaginal exposure homework<br>. | 9-12 individual sessions<br>lasted 90 –120 min. once a week | 5 female clinicians with doctoral degrees in clinical psychology at the academic clinic, and 6 clinicians with master's degrees in counseling or social work at the community clinic, receiving 2x5 days training and ongoing supervision | Wait-list: 1x phone-call of therapist to determine their state. Patients could call the therapist any time if they were having problems. | 9 weeks | A therapist |
| 6b [42] | Prolonged exposure in vivo plus cognitive restructuring: as condition 1 plus rationale that posttrauma symptoms are maintained in part by trauma-related<br>thoughts and beliefs and to practicing Cognitive Restructuring.(CR) all sessions included 30 – 45 min of imaginal exposure followed by 15–25 min of CR. | 9-12 individual sessions<br>lasted 90 –120 min. once a week | 5 female clinicians with doctoral degrees in clinical psychology at the academic clinic, and 6 clinicians with master's degrees in counselling or social work at the community clinic, receiving 2x5 days training and ongoing supervision | Wait-list: 1x phone-call of therapist to determine their state. Patients could call the therapist any time if they were having problems. | 9 weeks | A therapist |
| 7 [38] | Experimental condition 1: Exposure in vivo: multiple sessions that consisted of a gradual return to real-life settings that reminded the patient of the trauma/accident. The setting provoked increasing levels of anxiety over time | Multiple sessions during several weeks- months | Psychologist | Imaginal (prolonged) exposure consisted of relaxation and 4 sessions of imaginal exposure concerning 4 scenes related to the day of the accident | Multiple sessions | Psychologist |
