# Supplementary material for: Exposure-in-vivo containing interventions to improve work functioning of workers with anxiety disorder: a systematic review
Source: BMC Public Health. 2010 Oct 11;10:598. doi: 10.1186/1471-2458-10-598 (PMC3224747; doi:10.1186/1471-2458-10-598)
Supplement: Additional file 4 — For seven included studies and one meta-analysis the judgement of quality of evidence based on five GRADE criteria. For each study the study number, comparison a/b, and reference, first author, publication year, design (RCT/CT), and the concluding judgement of the quality of evidence based on the following five GRADE criteria: 1. conclusion of risk of bias transformed in GRADE-assessment of study limitation, 2. indirectness of evidence, 3. unexplained heterogeneity or inconsistency of results, 4. imprecision of results, 5. high probability of publication bias, are presented. [file 1471-2458-10-598-S4.PDF]

**Study number and reference**	**First author Publication year Design (RCT/CT)**	**1. Conclusion risk of bias transformed in GRADE-assessment of study limitation**	**2. Indirectness of evidence**	**3.Unexplained heterogeneity or inconsistency of results**	**4. Imprecision of results**	**5. High probability of publication bias**	**Conclusion: quality of evidence**
1 [37]	**Aigner (2004)** (CT)	High risk of bias: very serious limits[1]	No	No, based on one study	No	Unlikely	Low
2 [39]	**Greist (2002)** (RCT)	Unclear risk of bias: serious limits[2]	No	No, based on one study	No	Unlikely	Moderate
3 [41]	**Foa (1984)** (CT)	Unclear risk of bias: serious limits[3]	No	No, based on one study	No, N < 400 and effect size < 0.2	Unlikely	Moderate
4 [43]	**Marks (1988)** (RCT)	Low risk of bias: no serious limits[4]	No	No, based on one study	No, No outcome data available	Unlikely	High
5 [40]	**Cobb (1980)** (RCT)	Unclear risk of bias: Serious limits[5]	Yes (mixed group OCD +	No, based on one study	No, No outcome data available	Unlikely	Moderate

[1] A high risk of bias which means very serious limits for the quality of evidence (down two levels) due to no sequence generation (not randomized), no allocation concealment (allocation by preference of participants), no blinding of participants, incomplete outcome data not adequately adressed (12 dropouts), unclear selective outcome reporting (incomplete data reported), no other sources of bias.

[2] Unclear risk of bias which means serious limits for the quality of evidence (down one level) due to unclear sequence generation (randomized, but no further information), no allocation concealment (if exclusion then crossover to controls), blinding of interviewers and outcome-assessment, but incomplete blinding of participants, incomplete outcome data not adequately addressed (dropouts greater in computer-guided group then relaxation group; however no baseline differences), unclear selective outcome reporting, no other sources of bias.

[3] Unclear risk of bias which means serious limits for the quality of evidence (down one level) due to no random sequence generation (serial assignment procedure), no allocation concealment, blinding of assessors but not patients, incomplete outcome data adequately addressed, unclear selective outcome reporting, no other sources of bias.

[4] Low risk of bias which means no serious limits for the quality of evidence (down no level) due to an unclear sequence generation (randomized but no further information), unclear allocation concealment (no information available), double blinding of assessors however no further information available, incomplete outcome data adequately addressed, unclear selective outcome reporting, and no other sources of bias.

			severe phobia				
6 [42]	**Foa et al (2005)** RCT	Low risk of bias: no serious limits[6]	No	No, based on one study	No	Unlikely	High
7 [38]	**Salyards (2005)** CT	High risk of bias: very serious limits[7]	No (specific subgroup: PTSD non-visual flash backs)	No, based on one study	No	Unlikely	Low
8 [37,41]	**Meta-analysis (Comparison 1b + 3b)**	Unclear risk of bias: serious limits[8]	No	No	No	Unlikely	Moderate

---

5 Unclear risk of bias which means serious limits for the quality of evidence (down one level) due to unclear sequence generation (no information available) and unclear allocation concealment (no information available), no blinding, incomplete outcome data adequately addressed, unclear selective outcome reporting, and no other sources of bias.

6 Low risk of bias which means no serious limits for the quality of evidence (down no level) due to unclear sequence generation (a weighted randomization; no further information available), unclear allocation concealment (no information available), blinding of assessors, not patients, incomplete outcome data adequately addressed, unclear selective outcome reporting, and no other sources of bias.

7 High risk of bias which means very serious limits for the quality of evidence (down two levels) due to no sequence generation (not randomized), no allocation concealment (therapist judged allocation and allocation by preference of patients), no blinding of assessors, incomplete outcome data adequately addressed (no drop outs), unclear selective outcome reporting, and no other sources of bias.

8 Unclear to a high risk of bias which means serious to very serious limits for the quality of evidence (down one or two levels) due to no sequence generation (both studies not randomized), no allocation concealment in both studies, blinding in one study and no blinding in the other, incomplete outcome data not adequately addressed in both studies, unclear selective outcome reporting in both studies, and no other sources of bias in both studies.
