# Supplementary material for: Exposure-in-vivo containing interventions to improve work functioning of workers with anxiety disorder: a systematic review
Source: BMC Public Health. 2010 Oct 11;10:598. doi: 10.1186/1471-2458-10-598 (PMC3224747; doi:10.1186/1471-2458-10-598)
Supplement: Additional file 5 — For seven included studies containing 11 comparisons and one meta-analysis the effects on the work-related outcomes of workers with OCD and PTSD. For each comparison the study number, comparison a/b, and reference, quality of evidence, anxiety disorder, experimental group with exposure in vivo, control group without exposure in vivo, the effect-size of the work-related effect in SMD or OR, its 95% confidence interval, the reported test statistics, and p-value, are presented, as far as data were available. [file 1471-2458-10-598-S5.PDF]

Study					Work-related outcome					
**Number, comparison a/b, and reference**	**Quality of evidence (high, moderate, low)**	**Anxiety disorder**	**Experimental group with exposure in vivo**	**Control group without exposure in vivo**	**Effect of exposure vs non-exposure (significant positive=sp/ not significant=ns/ significant negative=sn). Effect size in standardised mean difference of follow-up (SMD) or Odds Ratio (OR) and its 95% confidence interval, reported test statistics, and p-value; .. = data not available**					
					**sp/ns/sn**	**SMD**	**OR**	**Confidence interval**	**Test statistics**	**p-value**
**1a [37]**	**low**	**OCD**	Group CBT	Medication (SSRIs)	sp	1.02	..	0.48, 1.55	..	..
**1b [37]**	**low**	**OCD**	Group CBT + medication	Medication (SSRIs)	sp	0.73	..	0.25, 1.20	..	..
**2a [39]**	**moderate**	**OCD**	Computer CBT (home via telephone)	Systematic self-relaxation	ns	0.35	..	0.08, 0.79	..	..
**2b [39]**	**moderate**	**OCD**	Clinician CBT private	Systematic self-relaxation	sp	0.72	..	0.28, 1.17	..	..
**3a [41]**	**moderate**	**OCD**	Exposure at home	Response prevention	ns	0.12	..	-1.02, 1.27	..	..
**3b [41]**	**moderate**	**OCD**	Exposure at home + response prevention	Response prevention	ns	0.68	..	-0.43, 1.79	..	..

**4 [43]**	**high**	**OCD**	Clomipramine with exposure homework	Clomipramine with anti-exposure homework	sp[1]	..	..	..	One-way ANOVA	0.03
**5 [40]**	**moderate**	**OCD / phobias**	Exposure therapy	Marital therapy	ns	..	..	..	Unpaired t-test	..
**1b+3b [37,41] Net contribution of exposure in vivo**	**moderate**	**OCD**	Exposure + (another) intervention X	Intervention X	sp	0.72	..	0.28, 1.15	Meta analysis Fixed effects	0.05
**6a [42]**	**high**	**PTSD**	Prolonged exposure	Wait-list	sp[2]	0.82	..	0.12, 1.52	Unpaired t-test	<0.01
**6b [42]**	**high**	**PTSD**	Prolonged exposure + cognitive restructuring	Wait-list	sp[2]	0.77	..	0.02, 1.51	Unpaired t-test	<0.01
**7 [38]**	**low**	**PTSD**	Exposure in vivo	Imaginal exposure	ns[3]	..	1.27	0.49, 3.31	Chi-square test	0.63

[1] On the self-rated work-related change score (group × time effect) we found a significant positive effect; on the assessor-rated work-related change score we found no significant effect.

[2] No follow up data were available for the control group; only post treatment data.

[3] Based on dichotomous return to work data: return to work or no return to work.
