# Supplementary material for: Exposure-in-vivo containing interventions to improve work functioning of workers with anxiety disorder: a systematic review
Source: BMC Public Health. 2010 Oct 11;10:598. doi: 10.1186/1471-2458-10-598 (PMC3224747; doi:10.1186/1471-2458-10-598)
Supplement: Additional file 6 — For seven included studies containing 11 comparisons and one meta-analysis the effects on the anxiety-related outcomes of workers with OCD and PTSD, are presented. For each comparison the study number, comparison a/b, and reference, quality of evidence, anxiety disorder, experimental group with exposure in vivo, control group without exposure in vivo, the effect-size of the anxiety-related effect in SMD or OR, its 95% confidence interval, the reported test statistics, and p-value, are presented, as far as data were available. [file 1471-2458-10-598-S6.PDF]

Study					Anxiety-related outcome				
**Number, comparison a/b, and reference**	**Quality of evidence (high, moderate, low)**	**Anxiety disorder**	**Experimental group with exposure in vivo**	**Control group without exposure in vivo**	**Effect of exposure vs non-exposure (significant positive=sp/not ignificant=ns/significant negative=sn). Effect size in standardised mean difference of follow-up (SMD) and its confidence interval, reported test statistics, and p-value; .. = data not available**				
					**sp/ns/sn**	**SMD**	**Confidence interval**	**Test statistics**	**p-value**
**1a [37]**	**high**	**OCD**	Group CBT	Medication (SSRIs)	sp	0.87	0.34, 1.39	..	..
**1b [37]**	**high**	**OCD**	Group CBT + medication	Medication (SSRIs)	sp	1.00	0.52, 1.49	..	..
**2a [39]**	**moderate**	**OCD**	Computer CBT (home via telephone)	Systematic self-relaxation	sp	0.72	0.28, 1.17	..	..
**2b [39]**	**moderate**	**OCD**	Clinician CBT private	Systematic self-relaxation	sp	1.01	0.55, 1.47	..	..
**3a [41]**	**moderate**	**OCD**	Exposure at home	Response prevention	ns (15×)[4]<br>Conclusion: ns<br>Obsessions	 <br>-0.52	 <br>-1.69, 0.64	..	..

					(self-rated)<br>Compulsions	0.15<br>Others (13x) available on request	-0.99, 1.30<br>Others (13x) available on request		
**3b [41]**	**moderate**	**OCD**	Exposure at home + response prevention	Response prevention	sp[5] (1x) severity of rituals and ns (14x). Conclusion: ns<br>Obsessions (self-rated)<br>Compulsions	-0.03<br>0.21<br>others (12x) available on request	-1.10, 1.04<br>-0.86, 1.29<br>others (12x) available on request	..	..
**4 [43]**	**high**	**OCD**	Clomipramine with exposure	Clomipramine with anti-exposure	sp[6] (9×)<br>ns (5×)	..	..	One-way ANOVA	Range 0.03–0.0001

			homework	homework	Conclusion: sp				
**5 [40]**	**moderate**	**OCD/ Phobias**	Exposure therapy	Marital therapy	sp[7] (1x) ns (3x)	..	..	Unpaired t-test	0.05
**1b+3b [37, 41] Net contribution of exposure in vivo**	**moderate**	**OCD**	Exposure + (another) Intervention X	Intervention X	ns	0.54	-0.16, 1.24	Meta analysis random effects	0.05
**6a [42]**	**high**	**PTSD**	Prolonged exposure	Wait-list	sp	1.92	1.35, 2.49	Unpaired t-test	<0.001
**6b [42]**	**high**	**PTSD**	Prolonged exposure + cognitive restructuring	Wait-list	sp	1.80	1.22, 2.38	Unpaired t-test	<0.001
**7 [38]**	**low**	**PTSD**	Exposure in vivo	Imaginal exposure	ns[8] (1x) Others (3x) on request	-0.11 Others (3x) on request	-0.55, 0.33 Others (3x) on request	Single effects test	0.51

[4] On fifteen outcome measures we found no significant effect: exposure test, assessor- and self-rated main fear, assessor- and self-rated avoidance, assessor- and self-rated obsessions, self-monitored rituals, assessor- and self-rated severity of rituals, assessor-rated urges to ritualize, MOC washing, compulsion checklist, and assessor- and self-rated anxiety.

[5]On one out of the fifteen outcome measures we found a positive significant effect: assessor-rated severity of rituals. On the other fourteen outcome measures mentioned at [6] we found no significant effect.

[6] On nine out of fourteen outcome measures we found a significant positive effect: assessor and self-rated time spent on four target rituals, assessor-rated discomfort of four target rituals, assessor- and self-rated total daily time spent on rituals, assessor- and self-rated global severity of rituals, self-rated severity of obsessive thoughts, and compulsion checklist. On the other five outcome measures we found no significant effect: self-rated discomfort of four target rituals, assessor-rated severity of obsessions, the self- and assessor-rated daily time spent on obsessive thoughts, and self-rated behavioural avoidance test.

[7] On one out of four outcome measures we found a positive significant effect: two main phobic–obsessive target problems. On the other three outcome measures we found no significant effect: Phobias and obsessions (self- and assessor-rated), Fear Survey Schedule. OC patients: two main obsessions (daily time taken up or discomfort), OC checklist.

[8] PTSD symptoms.
